# From online to offline: the impact of digital communication on activity participation among older adults

**DOI:** 10.3389/fpubh.2026.1804827

**Published:** 2026-04-30

**Authors:** Geng Wang

**Affiliations:** School of Journalism & Communication, Liaoning University, Shenyang, China

**Keywords:** Chinese older adults, digital communication, offline activity participation, social self-efficacy, social capital, moderated mediation model

## Abstract

**Objectives:**

At the nexus of rapid digitization and demographic aging, leveraging the social potential of digital technologies to achieve active aging goals has emerged as a key concern. Drawing on the digital empowerment perspective, this research empirically examined the association between digital communication and older adults’ offline activity participation, while further investigating the mediating roles of three types of social capital and the moderating role of social self-efficacy.

**Methods:**

We conducted a cross-sectional survey administered offline and collected 1,595 valid responses (mean age = 66.01 years). The survey assessed older adults’ digital communication use, frequency of offline activity participation, three types of social capital, social self-efficacy, and key demographic characteristics. SPSS along with Hayes Process Models 4 and 7 was employed to test the hypotheses.

**Results:**

(1) Digital communication was significantly and positively associated with older adults’ offline activity participation. (2) Bonding, bridging, and maintained social capital each mediated the association between digital communication and offline activity participation. (3) Social self-efficacy negatively moderated the associations between digital communication and three types of social capital, such that the positive association was stronger among older adults with lower social self-efficacy.

**Conclusion:**

For older adults, digital communication extends beyond serving merely as an informational or interactive tool. Through its associations with multidimensional social capital, it is closely intertwined with offline participation, lending empirical support to the network enhancement effect in the context of population aging. The more pronounced compensatory pattern observed among older adults with lower self-efficacy further suggests that digital communication may be particularly relevant to the social integration of psychologically vulnerable groups. Accordingly, we recommend that age-friendly digital initiatives prioritize older adults’ perceived digital benefits, strengthen the pathways linking online interaction to offline participation, and provide tailored technological support for older adults with different personality traits that aligns with their sustained motivation for social engagement.

## 1 Introduction

The data indicate that by the end of 2024, China’s population aged 60 and above exceeded 310 million (22.0% of the total), while those aged 65 and above surpassed 220 million (15.6%), signaling that China has entered a critical stage of moderate population aging (1). Against this backdrop, active aging has become a key public policy response to population aging (2). It emphasizes older adults’ autonomy and agency, while promoting continued social engagement and contribution in later life as a means of enhancing well-being (3, 4). The realization of these policy objectives hinges on whether older adults can remain involved in public life and sustain face-to-face social interactions in their daily lives (5, 6). Accordingly, offline activity participation has emerged as one of the core concepts for capturing the practical foundations of active aging (7). It refers to older adults’ sustained involvement in community and broader public life within physical locales, instantiated primarily through face-to-face interactions. Offline activity participation can be defined by three core features: it is driven by individuals’ willingness, capacities, and preferences and takes place across a broad range of public settings; it includes not only relatively organized formal activities, such as cultural and sporting events, leisure pursuits, community governance, and volunteering, but also socially oriented informal offline interactions and collective activities; and it emphasizes sustained engagement over time rather than occasional outings (8–10). Offline activity participation not only serves as a direct indicator of older adults’ social integration and role continuity, but it also functions as a protective strategy for mitigating the risks of role loss and the erosion of social ties (11–13). By facilitating the co-creation of personal and social value across psychosocial and material domains, it helps unlock the longevity dividend and underpins the sustainability of the aging society.

As population aging deepens, Chinese society is simultaneously witnessing rapid advances in digital and intelligent technologies. Digital media have become pervasively and profoundly embedded in older adults’ everyday lives, reshaping their patterns of social connectedness (14, 15). By June 2025, the number of Chinese Internet users aged 60 and above reached 161 million, with a penetration rate of 52.0% (16). Older adults’ engagement with digital media, encompassing initial access, sustained connectivity, and routine use, has evolved from fragmented, marginal experimentation into a normalized practice. However, existing research on how older adults’ use of digital media affects their physical health, mental health, and social integration has yielded divergent conclusions (17, 18). Some studies underscore its potential to enhance well-being (19–21), whereas others draw attention to its associated risks (22–24), resulting in a mixed and often contradictory evidence base regarding its benefits and harms. In addition, within the framework of active aging, there remains a lack of empirical evidence regarding the specific effects of digital media use on older adults’ offline participation. A limited number of studies have examined older adults’ physical exercise as an outcome variable, yet the findings remain mixed: digital media may promote participation in physical exercise by facilitating information access and social interaction, but it may also suppress such participation through a time displacement effect (25, 26). Research suggests that these divergent findings may be partly attributable to the fact that existing studies have not sufficiently distinguished among the specific purposes and functions of digital media use (27). In light of these gaps, the present study focuses specifically on the digital communication function, adopts a digital empowerment perspective, and employs a questionnaire-based survey design to examine whether and through which mechanisms digital media use is associated with older adults’ offline activity participation, as well as whether the strength of these associations varies across older adults with different personality traits. By addressing these concerns, the study aims to generate empirical evidence on how technological mediation empowers older adults’ digital lives.

## 2 Literature review and research hypotheses

### 2.1 Digital communication and offline activity participation

In scholarly discussions of the relationship between digital media use and older adults’ offline participation, two major theoretical perspectives have emerged: one emphasizing the network enhancement effect and the other highlighting the presence substitution effect (28, 29). The former posits that digital media provide older adults with a platform that is less constrained by time and space, enabling them to connect with broader and more diverse social networks (30, 31). Consequently, digital media broaden the scope of older adults’ social interactions and strengthen their capacity for information acquisition, thereby improving access to public information and social opportunities and increasing the likelihood that they will join social groups and engage in diverse forms of social participation (32–35). By contrast, the presence substitution perspective treats digital media use as a competing allocation of time and attention that may crowd out offline social participation or disrupt ongoing activities (36–38). On the one hand, as older adults spend more time online, the time, energy, and attentional resources available for community engagement and face-to-face interaction may be reduced, shifting leisure and entertainment toward more indoor and privatized forms and potentially increasing the risk of social withdrawal (39, 40). On the other hand, the convenience of digital media may prompt individuals to substitute low-investment virtual connections for high-involvement offline interactions, making relationship maintenance increasingly reliant on superficial exchanges and, to some extent, impeding the development of social connectedness, trust, and empathy in deeper interpersonal interactions (41, 42). This may, in turn, weaken strong ties characterized by care and intimacy (24, 43), undermine older adults’ sense of community belonging (44), and ultimately constrain their social integration (45, 46).

Previous research has suggested that a major source of divergent conclusions lies in the predominance of macro-level perspectives and the reliance on single, undifferentiated measures of digital media use, thereby obscuring the heterogeneous effects of different functional uses (17). Empirical evidence further indicates that older adults’ differential selection of media functions and variation in their usage patterns constitute key explanatory factors underlying heterogeneity in media effects (47). Accordingly, a critical step in understanding media effects and their boundary conditions is to identify what specific activities older adults engage in online by functionally disaggregating user behavior and examining the underlying mechanisms within clearly defined usage contexts (48). Consistent with this reasoning, the present study narrows its analytical focus to what is arguably older adults’ most central digital practice: the use of digital media for communication (49). As a prototypical form of supportive technology, digital communication is associated with older adults’ efforts to establish and maintain connections with the broader society (50). This function not only represents a key driver of their initial adoption of digital technology but also constitutes a prominent and enduring functional motivation underlying their continued use. It thus forms a foundational dimension of older adults’ digital practices (51, 52). The present study focuses on analyzing the relationship between this functional use and older adults’ offline participation, while further exploring the specific mechanisms underlying this association. Moreover, in deriving the direction of the hypothesis, we draw on the theory of selective optimization with compensation (53), viewing older adults as rational agents who make decisions under conditions of resource constraints and changing functional capacities. From this perspective, their use of the digital communication function is understood as a process of actively selecting and adaptively engaging with affordance-bearing communicative resources (54). Older adults tend to allocate their limited resources more efficiently, both to compensate for age-related developmental losses and to obtain the information and supportive resources needed for participation in social and public life (55). Thus, we propose the following research hypothesis.

*Hypothesis 1*: Digital communication use is positively associated with older adults’ offline social participation.

### 2.2 The mediating role of social capital

The concept of social capital was first systematically elaborated by the French sociologist Bourdieu in the 1980s (56). Some scholars define it as supportive social networks among citizens, together with the associated norms of reciprocity and relations of trust (57). At the micro level, social capital is commonly conceptualized as the resources embedded in relational ties and their potential for mobilization. It comprises the social relationships, resources accessible through these relationships (e.g., access to information, emotional support, instrumental support), and individuals’ capacity to convert these resources into tangible benefits (58). In brief, the notion of social capital employed in this study foregrounds its relational and resource dimensions, focusing primarily on the accessibility of resources provided through social networks. At the individual level, this manifests as a stable expectation of obtaining assistance when needed. This antecedent perception may underpin and facilitate individuals’ subsequent social behavior (59). Digital media, particularly the communicative functions, provide a critical technological foundation for the generation and accumulation of social capital. As highly efficient and convenient tools for social connection, digital communication technologies may enable older adults to maintain and expand their social relationships through affordances for transcending temporal and spatial barriers and reducing interaction costs (60). First, within strong-tie relationships, digital communication can foster high-frequency interactions with family members and close friends, thereby reinforcing bonding social capital grounded in resource provision and emotional support (61, 62). Second, within weak-tie networks, digital communication creates opportunities for contact that transcend social and geographic distance (63). In doing so, it enables older adults to obtain heterogeneous resources and nonredundant information from relatively loose-knit networks, and to forge connections with a wider range of communities, thereby fostering bridging social capital (64). Third, given the risk of social network contraction among older adults as they undergo life-course transitions such as relocation and retirement, digital communication provides a low-cost mechanism for maintaining social relationships. It allows previously dormant or disrupted ties to be reactivated and reinforced through ongoing interactions, thereby substantially increasing stocks of maintained social capital (63). Viewed collectively, these processes underscore that older adults’ use of digital communication functions is closely associated with their practices of coping with social isolation and with the construction of diverse forms of social capital.

Furthermore, there is a close association between social capital and older adults’ offline activity participation. Within the social contagion framework, dense networks of strong ties are understood to function as powerful conduits for behavioral diffusion (65–67). For older adults embedded in these networks, processes of peer modeling and interactional reinforcement cultivate a stronger propensity to engage in offline activities. Existing research further indicates that a rich stock of social capital not only improves older adults’ access to activity-related information and lowers barriers to offline participation, but also furnishes instrumental support, including accompaniment and practical assistance, that reduces concrete hurdles to engaging in social activities (68, 69). At the same time, expectations of reciprocity and foundations of trust within social networks substantially diminish the uncertainty surrounding interpersonal interactions (70), thereby providing older adults with a sense of psychological security for engaging in community life. This sense of security is, in turn, associated with both the likelihood and frequency of their offline participation. Accordingly, the present study posits that social capital serves as a key explanatory mechanism linking digital communication to older adults’ offline activity participation. On this basis, the following research hypotheses are advanced.

*Hypothesis 2a*: Bonding social capital mediates the association between digital communication use and offline activity participation.

*Hypothesis 2b*: Bridging social capital mediates the association between digital communication use and offline activity participation.

*Hypothesis 2c*: Maintained social capital mediates the association between digital communication use and offline activity participation.

### 2.3 The moderating role of social self-efficacy

**Figure 1:**
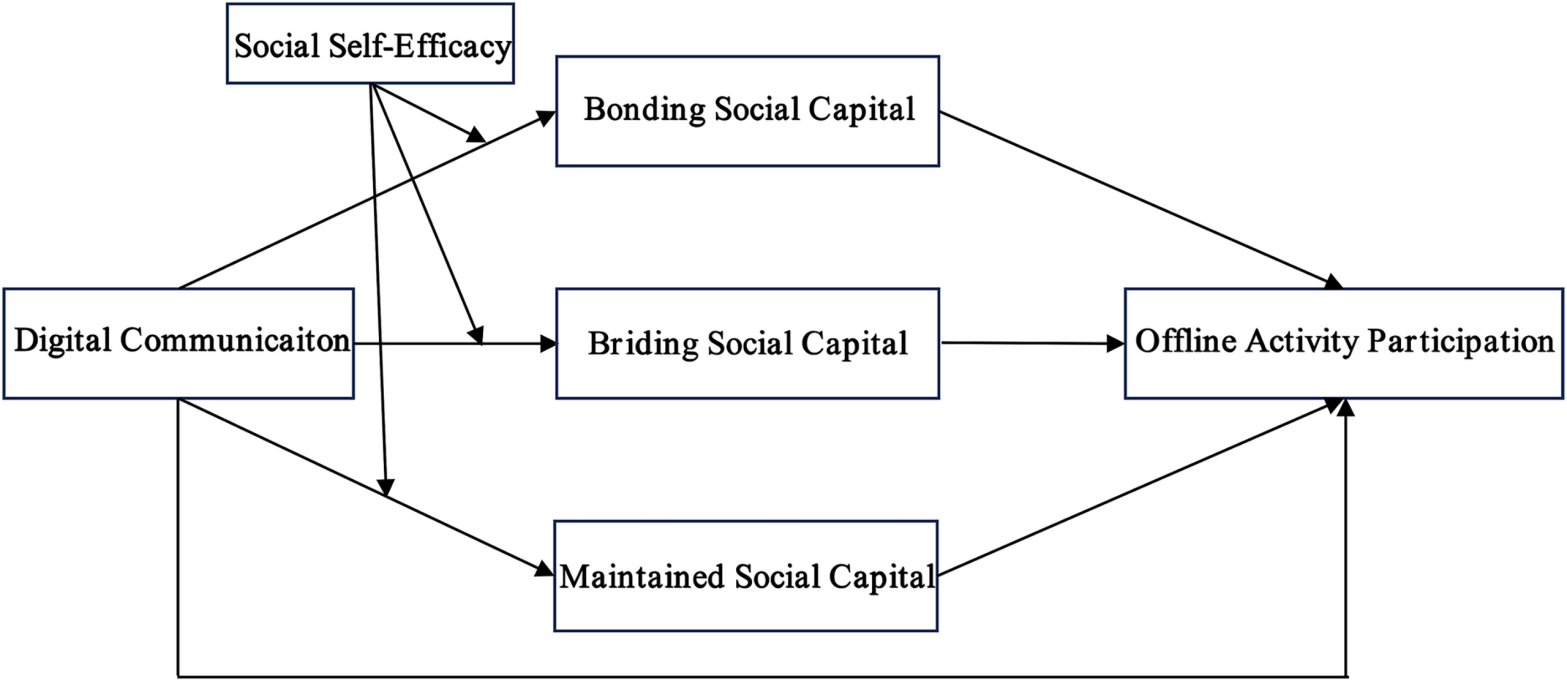
The research model.

Given the substantial and growing size of the older population, this study views older adults as an internally diverse population whose differences cannot be adequately captured by simple demographic labels but are reflected in marked variation in personality traits and psychological characteristics (71). Although digital media may provide older adults with opportunities to accumulate social capital, individual psychological attributes are likely to function as key boundary conditions of media effects, shaping the extent to which they derive benefits from digital communication practices (72–74). The foregoing discussion aligns with competing hypotheses in the existing literature regarding who is more likely to benefit from online social interactions. On the one hand, the social enhancement (rich get richer) hypothesis holds that individuals with high social self-confidence and strong interpersonal skills are better able to leverage digital media as a functional extension of their capacities for relationship management and resource mobilization, thereby further consolidating and expanding their existing social networks and attaining higher levels of social capital (75–77). On the other hand, the social compensation (poor get richer) hypothesis holds that digital media confer greater marginal benefits on individuals who are introverted and less socially skilled (78). It argues that virtual environments provide a compensatory channel for those who are disadvantaged in offline social interactions. By virtue of features such as anonymity and non-face-to-face communication, digital media reduce psychological pressure associated with interpersonal encounters, enabling socially anxious individuals to communicate, obtain support, and form social ties under relatively comfortable conditions. In this way, digitally mediated interactions are, to some extent, associated with compensation for deficits in their offline social lives and with gains in social capital (63, 79, 80). It should be noted that empirical evidence for these two hypotheses has been derived primarily from adolescent and general adult samples (24, 81, 82), with little in-depth investigation conducted in the context of population aging, particularly among older adults. To clarify which of the competing hypotheses better accounts for the outcomes of older adults’ digital practices, this study incorporates social self-efficacy as a moderating variable. Social self-efficacy is used to capture older adults’ beliefs in their ability to initiate and establish friendships in social situations, as well as their confidence in performing the interaction tasks required to maintain interpersonal relationships (83, 84). Specifically, the study examines whether variation in social self-efficacy, an important internal psychological attribute of older adults, moderates the strength of the association between digital communication and the accumulation of social capital, thereby illuminating whether, for older adults, digital communication operates primarily as a mechanism of cumulative advantage or as a compensatory pathway for pre-existing resource deficits. Guided by the above considerations, this study proposes the following research question and develops the research model depicted in Figure 1.

*Research question*: How does older adults’ social self-efficacy moderate the association between digital communication use and the accumulation of social capital?

## 3 Materials and methods

### 3.1 Participants

Given regional disparities in China’s Internet development and the uneven spatial distribution of population aging (85, 86), we conducted extensive on-site intercept sampling across eastern, central, and western China. Participants were eligible if they met the statutory retirement age criterion (males ≥60 years; females ≥55 years). In selected cities, participants were recruited on-site from settings in which older adults’ daily activities were relatively concentrated, including residential communities, public activity centers, and marketplaces. In total, 1,887 questionnaires were collected. To safeguard data quality, we excluded invalid responses due to age ineligibility, misunderstanding of the definition of digital media, more than 20% missing responses, failure to pass attention checks, and evidence of patterned responding. The final analytic sample consisted of 1,595 valid responses, yielding a valid response rate of 84.53%. Demographic characteristics of the sample are reported in Table 1. Overall, 87.21% of respondents indicated access to digital media, which exceeded the national estimate of Internet access among older adults reported by CNNIC (16). This difference may be attributable to the study’s sampling design and measurement scope. First, the sample consisted of urban older adults, who generally had better access to information and communication technologies (87). Second, to maintain response quality, we excluded individuals aged 85 and above, as cognitive or physical constraints were more likely in this age group and could compromise reliable survey completion (88). Third, we defined digital media broadly to encompass multiple smart devices and use contexts, such as smartwatches and smart speakers, rather than limiting measurement to conventional Internet use, thereby capturing a more inclusive range of digital access. Accordingly, the sample was appropriate for the analyses within the intended scope of the study and provided adequate explanatory leverage for our research questions.

**Table 1:** Demographic information of the participants.

Characteristics	Categories	Number	Mean ± SD/percentage
Gender	Female	1,047	65.64%
	Male	548	34.36%
Age	T	1,595	66.01 ± 7.31
Educational level	Primary school or below	543	34.04%
	Junior high school	467	29.28%
	High school	274	17.18%
	Junior college	109	6.83%
	Bachelor’s degree or higher	202	12.66%
Marital status	In marital status	1,202	75.36%
	Not in marital status	393	24.64%
Self-rated health	Very unhealthy	112	7.02%
	Somewhat unhealthy	255	15.99%
	Moderately healthy	848	53.17%
	Fairly healthy	307	19.25%
	Very healthy	73	4.58%
Region of residence	Eastern region	491	30.78%
	Central region	524	32.85%
	Western region	580	36.36%
Digital media access	With access	1,391	87.21%
	Without access	204	12.79%

### 3.2 Measures

#### 3.2.1 Digital communication use

Building on the function-based measurement approach to Internet use proposed by Lifshitz et al. (17) and Park et al. (89), this study examined the frequency of digital communication function use among older adults. A three-item scale was adapted to assess this behavior (e.g., “I communicate with friends online about certain topics”). Participants rated the frequency of these behaviors on a five-point Likert scale (1 = never, 5 = very frequently), with higher mean scores reflecting more frequent use. The scale demonstrated acceptable internal consistency (Cronbach’s *α* = 0.843). Notably, the questionnaire began by asking whether respondents had ever used any form of digital media. Respondents who reported no prior use were instructed to skip the three communication-use items. During preprocessing, these respondents were retained as valid cases and coded as 1 (“never used”) rather than treated as missing values, because nonuse represented a substantively meaningful response category indicating the absence of digital communication use.

#### 3.2.2 Offline activity participation

Following Baum et al.’s approach to measuring informal social participation, participation in public spaces, and group activities (90), this study adopted a four-item scale to assess older adults’ offline activity participation, excluding profit-oriented and work-related activities. Example items included “I frequently take part in organized leisure, cultural, or physical activities (e.g., mahjong, square dancing, group trips)” and “I frequently visit friends or attend social gatherings.” Respondents rated each item on a five-point Likert scale, with higher mean scores indicating more frequent participation. In the present study, the scale demonstrated excellent internal consistency (Cronbach’s *α* = 0.915).

#### 3.2.3 Social capital

To operationalize the construct of social capital, we drew on two widely used scales developed by Williams (91) and Ellison et al. (63). Given older respondents’ potential limitations in attention and comprehension, we employed a parsimonious measurement instrument that nonetheless captured the core dimensions. Specifically, two items were used to assess bonding social capital (e.g., “When I feel lonely, there are several people I can talk to”), and two items were used to assess bridging social capital (e.g., “In my interactions with others, I feel that I am part of a larger community”). For maintained social capital, we recognized that older adults differed from the student population for whom Ellison et al.’s original scale was developed (63). Among older adults, the need to maintain social capital arises primarily from disruptions to social relationships caused by retirement-related role exit or geographic relocation. We therefore adapted the items to the later-life context, focusing on older adults’ ability to reactivate or sustain pre-existing social ties through interpersonal communication (e.g., “If necessary, I can ask a former colleague to do me a small favor”). Respondents rated all items on a five-point Likert scale (1 = strongly disagree, 5 = strongly agree). Given that three types of social capital were each measured using two-item scales, we assessed internal consistency reliability by computing the inter-item Pearson correlations and the Spearman–Brown split-half reliability coefficients (92). The inter-item correlations for bonding, bridging, and maintained social capital were 0.837, 0.871, and 0.768 (*p* < 0.01). The corresponding Spearman–Brown coefficients were 0.911, 0.931, and 0.869. All indices exceeded the 0.7 threshold, demonstrating robust reliability for the mediators.

#### 3.2.4 Social self-efficacy

To assess older adults’ social self-efficacy, we drew on a subscale from Sherer et al.’s (93) self-efficacy scale. Considering older adults’ cognitive load and comprehension constraints, we selected two positively worded items from this subscale to assess their perceived ability to establish and maintain interpersonal relationships (e.g., “I have acquired my friends through my personal abilities at making friends”). Respondents rated each item on a five-point Likert scale (1 = strongly disagree, 5 = strongly agree), with higher mean scores indicating higher levels of social self-efficacy. The inter-item correlation was 0.788, and the corresponding Spearman–Brown coefficient was 0.881.

#### 3.2.5 Control variables

This study additionally controlled for a set of individual characteristics, including gender, age, educational level, marital status, self-rated health, and region of residence. Prior research has shown that each of these factors is significantly associated with older adults’ offline activity participation (94–96).

### 3.3 Procedure

This study employed a cross-sectional, face-to-face survey design. Prior to the main survey, we conducted a pilot survey with 20 older adults who completed the questionnaire on-site and then participated in item-by-item debriefing interviews about their interpretation of each question. The pilot indicated that some older adults had substantial difficulty interpreting reverse-worded items and experienced elevated cognitive load when responding to longer scales. Based on these findings, we restricted both the number and linguistic complexity of items when selecting and adapting the relevant scales, retaining a concise set of positively phrased items with clear, straightforward content to reduce response burden for older adults. Subsequently, the main survey was administered by 10 undergraduate interviewers who had received standardized training. In accordance with ethical guidelines, interviewers clearly explained the study purpose to all potential participants, clarified the meaning of the core construct “digital media,” and emphasized both the anonymity of participation and participants’ right to withdraw at any time without penalty. Questionnaires were distributed only after written informed consent had been obtained, and interviewers were present to provide on-site assistance during completion. To assess respondents’ basic understanding of the concept of digital media, two concept-check items were placed on the first page of the questionnaire, asking whether a smart speaker and a printed newspaper should be considered forms of digital media. During data screening, any questionnaire in which either item was answered incorrectly was classified as invalid and excluded from subsequent analyses. All questionnaires were entered into SPSS 27.0 by two independent research assistants, and discrepancies were identified and resolved through consistency checks. The study protocol was approved by the institutional ethics review board.

### 3.4 Data analysis

Statistical analysis was primarily conducted using SPSS 27.0 and the PROCESS macro 4.0. A three-stage analytical framework was adopted to rigorously test the research hypotheses. In the first stage, a confirmatory factor analysis (CFA) was implemented to evaluate the measurement model, with a primary emphasis on establishing the empirical distinctiveness of the three mediators (bonding, bridging, and maintained social capital). In the second stage, a mediation model was assessed via Hayes’ (97) Model 4. In the third stage, the moderating effect of social self-efficacy on the indirect pathway was examined using Model 7. The significance of the moderated mediation model was tested using bootstrapping with 5,000 resamples and 95% confidence intervals to assess the stability of the indirect effects.

## 4 Results

### 4.1 Preliminary analyses: descriptive statistics, measurement validity, and multicollinearity diagnostics

Descriptive statistics and correlations for the core variables are summarized in Table 2. All six variables exhibit significant pairwise associations (*p* < 0.01). Given that several key constructs were measured using two-item scales and that the three social capital mediators showed relatively strong intercorrelations, we conducted additional checks at both the measurement and estimation levels. First, we reported inter-item correlations for all two-item measures as a direct indicator of internal consistency. We then conducted a confirmatory factor analysis (CFA) on the full measurement model including all 15 items. The CFA indicated acceptable model fit (CFI = 0.969, TLI = 0.957, RMSEA = 0.071, SRMR = 0.043), and all standardized factor loadings were statistically significant and exceeded the recommended threshold of 0.7, supporting convergent validity. Convergent validity was further supported by composite reliability (CR) and average variance extracted (AVE), with CR values exceeding 0.70 and AVE values exceeding 0.50 for all constructs. Discriminant validity was supported by the Fornell–Larcker criterion (98), as the square root of AVE for each construct exceeded its correlations with other constructs, indicating the empirical distinctiveness of the three types of social capital. Finally, to address concerns that high correlations among the mediators might inflate standard errors, we reported variance inflation factors (VIFs). All VIF values were below 3, indicating no serious multicollinearity. Together, these results provided a sound measurement and estimation basis for the subsequent moderated mediation analyses. These results are all presented in Table 2.

**Table 2:** Means, standard deviations, correlations, and reliability/validity indices (*N* = 1,595).

Variables	Mean	SD	CR	AVE	VIF	1	2	3	4	5	6
1. Offline activity participation	3.273	0.943	0.924	0.755	—	(0.869)					
2. Digital communication use	3.128	1.230	0.915	0.783	1.192	0.394^**^	(0.885)				
3. Bonding capital	3.707	0.865	0.911	0.837	2.124	0.508^**^	0.336^**^	(0.915)			
4. Bridging capital	3.343	1.000	0.931	0.871	1.597	0.524^**^	0.356^**^	0.566^**^	(0.933)		
5. Maintained capital	3.524	0.876	0.869	0.769	1.886	0.463^**^	0.316^**^	0.659^**^	0.501^**^	(0.877)	
6. Social self-efficacy	2.898	0.918	0.892	0.808	1.158	0.260^**^	0.138^**^	0.351^**^	0.236^**^	0.316^**^	(0.899)

Diagonal elements (in parentheses) are the square roots of the AVE. **p < 0.01.

### 4.2 Mediation effect test

We conducted a parallel mediation analysis using Model 4 in SPSS PROCESS to examine the indirect associations between digital communication use and offline activity participation via social capital, with demographic characteristics included as covariates. As shown in Table 3, digital communication use was positively associated with older adults’ bonding capital (*Coeff* = 0.232, *p* < 0.001), bridging capital (*Coeff* = 0.265, *p* < 0.001), maintained capital (*Coeff* = 0.222, *p* < 0.001), and offline activity participation (*Coeff* = 0.134, *p* < 0.001). Thus, Hypothesis 1 was supported. Moreover, bonding capital (*Coeff* = 0.219, *p* < 0.001), bridging capital (*Coeff* = 0.250, *p* < 0.001), and maintained capital (*Coeff* = 0.146, *p* < 0.001) were positively associated with offline activity participation. The bias-corrected percentile bootstrap analysis showed that bonding capital [indirect effect = 0.051, 95% CI (0.032, 0.071)], bridging capital [indirect effect = 0.066, 95% CI (0.049, 0.086)], and maintained capital [indirect effect = 0.032, 95% CI (0.018, 0.050)] each partially mediated this association, accounting for 17.91, 23.34, and 11.41% of the total effect, respectively. Thus, Hypotheses 2a, 2b and 2c were supported.

**Table 3:** Results of the parallel multiple mediator model analysis.

Antecedents	Consequent															
	Bonding capital				Bridging capital				Maintained capital				Offline activity participation			
	*Coeff*	*β*	*SE*	*t*	*Coeff*	*β*	*SE*	*t*	*Coeff*	*β*	*SE*	*t*	*Coeff*	*β*	*SE*	*t*
Constant	2.033	—	0.240	8.465^***^	2.578	—	0.275	9.370^***^	2.419	—	0.246	9.836^***^	0.580	—	0.228	2.545^*^
Digital communication use	0.232	0.330	0.017	13.661^***^	0.265	0.326	0.019	13.610^***^	0.222	0.312	0.017	12.769^***^	0.134	0.175	0.017	8.023^***^
Bonding capital	—	—	—	—	—	—	——	—	—	—	—	—	0.219	0.201	0.031	7.082^***^
Bridging capital	—	—	—	—	—	—	——	—	—	—	—	—	0.250	0.265	0.024	10.569^***^
Maintained capital	—	—	—	—	—	—	—	—	—	—	—	—	0.146	0.135	0.029	5.040^***^
Control variables																
Gender	0.064	0.035	0.044	1.457	−0.002	−0.001	0.050	−0.032	−0.017	−0.009	0.045	−0.382	−0.015	−0.008	0.040	−0.380
Age	0.008	0.071	0.003	2.861^**^	−0.008	−0.055	0.003	−2.269^*^	0.005	0.039	0.003	1.577	−0.004	−0.031	0.003	−1.503
Educational attainment	0.020	0.031	0.016	1.254	0.062	0.084	0.018	3.420^***^	0.013	0.020	0.016	0.805	0.020	0.029	0.015	1.404
Marital status	0.003	0.001	0.049	0.056	0.043	0.019	0.056	0.770	−0.006	−0.003	0.050	−0.128	0.109	0.050	0.045	2.443^*^
Self-rated health status	0.084	0.087	0.023	3.657^***^	0.071	0.064	0.026	2.714^**^	0.072	0.075	0.023	3.099^**^	0.050	0.048	0.021	2.379^*^
Place of residence	−0.004	−0.004	0.025	−0.178	0.013	0.011	0.028	0.474	−0.054	−0.051	0.025	−2.138^*^	0.034	0.029	0.023	1.495
*R* ^2^	0.127				0.145				0.109				0.390			
*F*	32.992^***^				38.324^***^				27.847^***^				101.405^***^			

Coeff denotes unstandardized coefficients, whereas β denotes standardized coefficients. ^*^p < 0.05; ^**^p < 0.01; ^***^p < 0.001.

### 4.3 Moderated mediation effect test

**Figure 2:**
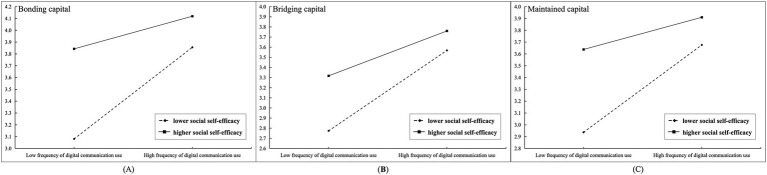
The moderating effects of social self-efficacy on the associations between digital communication use and three types of social capital. In **(A)**, the outcome variable is bonding capital. In **(B)**, the outcome variable is bridging capital, and in **(C)**, the outcome variable is maintained capital.

To test the moderating effect of social self-efficacy, Model 7 was then employed. As shown in Table 4, social self-efficacy significantly moderated the associations between digital communication use and bonding capital (*Coeff* = −0.111, *p* < 0.001), bridging capital (*Coeff* = −0.078, *p* < 0.001), and maintained capital (*Coeff* = −0.103, *p* < 0.001), indicating negative moderation effects in all three cases. Compared with older adults with higher social self-efficacy, the positive association between digital communication use and social capital was more pronounced among those with lower social self-efficacy, and this pattern was consistently observed across bonding, bridging, and maintained social capital. To visualize the moderated effects, we plotted simple slopes showing the associations between digital communication use and social capital at low (−1 SD) and high (+1 SD) levels of social self-efficacy in Figure 2. Moreover, conditional indirect effect analyses indicated that when social self-efficacy was set at three representative levels (low, mean, and high), the 95% bootstrap confidence intervals did not include zero, indicating that the corresponding mediating mechanisms were statistically significant across levels of social self-efficacy.

**Table 4:** Results of the moderated mediation model analysis.

Antecedents	Consequent											
	Bonding capital			Bridging capital			Maintained capital			Offline activity participation		
	*Coeff*	*SE*	*t*	*Coeff*	*SE*	*t*	*Coeff*	*SE*	*t*	*Coeff*	*SE*	*t*
Constant	2.971	0.218	13.638^***^	3.559	0.261	13.662^***^	3.307	0.226	14.616^***^	1.001	0.228	4.400^***^
Digital communication use	0.214	0.016	13.302^***^	0.252	0.019	13.103^***^	0.205	0.017	12.319^***^	0.134	0.017	8.023^***^
Social self-efficacy	0.279	0.021	13.255^***^	0.200	0.025	7.950^***^	0.255	0.022	11.649^***^	—	—	—
Bonding Capital	—	—	—	—	—	—	—	—	—	0.219	0.031	7.082^***^
Bridging Capital	—	—	—	—	—	—	—	—	—	0.250	0.024	10.569^***^
Maintained Capital	—	—	—	—	—	—	—	—	—	0.146	0.029	5.040^***^
Digital communication use * Social self-efficacy	−0.111	0.016	−7.119^***^	−0.078	0.019	−4.202^***^	−0.103	0.016	−6.414^***^	—	—	—
Control variables												
Gender	0.049	0.041	1.192	−0.012	0.049	−0.253	−0.031	0.042	−0.725	−0.015	0.040	−0.380
Age	0.007	0.003	2.530^*^	−0.009	0.003	−2.639^**^	0.003	0.003	1.203	−0.004	0.003	−1.503
Educational attainment	0.017	0.015	1.117	0.060	0.018	3.371^***^	0.010	0.015	0.653	0.020	0.015	1.404
Marital status	0.028	0.046	0.604	0.061	0.054	1.115	0.016	0.047	0.343	0.109	0.045	2.443^*^
Self-rated health status	0.047	0.022	2.195^*^	0.045	0.026	1.749	0.039	0.022	1.759	0.050	0.021	2.379^*^
Place of residence	0.001	0.023	0.026	0.017	0.028	0.616	−0.050	0.024	−2.064^*^	0.034	0.023	1.495
*R* ^2^	0.239			0.187			0.201			0.390		
*F*	55.188^***^			40.541^***^			44.270^***^			101.405^***^		

^*^p < 0.05; ^**^p < 0.01; ^***^p < 0.001.

## 5 Discussion

This study examined the association between digital communication use and older adults’ offline activity participation, as well as the mechanisms underlying this association. Using data from 1,595 valid questionnaires, we specified and tested a moderated mediation model to assess the mediating roles of three forms of social capital and the moderating role of social self-efficacy in these pathways. The main empirical findings are summarized as follows.

### 5.1 Network enhancement: the association between digital communication and offline activity participation

This study confirmed that the frequency of digital communication use was positively associated with older adults’ offline activity participation, consistent with Hypothesis 1. This finding underscores the distinctive character of digital communication relative to more one-way, consumption-oriented media practices such as information browsing and entertainment consumption. Oriented toward interactivity and relationship-building, digital communication may serve as a “low-barrier, highly accessible” mechanism for social connection (99). Its role extends beyond instantaneous information exchange, closely linking virtual interaction with offline embodied participation. In this sense, digital media use is associated with lower social isolation in later life. It may also help offset the constraints that residential distance and limited mobility place on offline participation, serving as both a technological resource and an empowering tool (73, 100, 101). Overall, the finding further substantiates the applicability of the network enhancement effect in the context of population aging. According to socioemotional selectivity theory, older adults who perceive their future time as limited exhibit pronounced selectivity in their social interactions and tend to invest their finite resources in social activities that provide stable emotional returns (102). For these individuals, digital communication is not merely a means of passing time or alleviating boredom, but a pragmatic medium oriented toward emotional fulfillment, relationship maintenance, and connections to offline social life (103). Their digital communication use appears to be strongly goal-directed, such that digital engagement is less likely to be associated with negative time-displacement effects. Instead, it may function as a facilitator of their integration into real-world community life.

Accordingly, contemporary initiatives to build age-friendly digital environments should prioritize optimizing communication functions, with particular emphasis on strengthening the links between digital spaces and everyday offline life. More specifically, digital technology-based support should be leveraged to lower older adults’ information search costs and decision-making barriers when accessing offline opportunities and resources. In parallel, digital literacy programs should strive to move beyond basic operational skills toward cultivating more advanced capacities for social conversion. Older adults should be encouraged to use digital tools more efficiently to obtain service and activity information as well as supportive resources, for instance, by using map applications to locate public cultural facilities or by proactively initiating offline gatherings within group chats. Such a synergistic mechanism, combining technological empowerment with enhanced digital literacy, may be associated with improvements in the quality of social participation and overall gains in well-being.

### 5.2 Digital empowerment: the mediating effect of social capital

This study demonstrated that social capital mediated the association between digital communication and older adults’ offline activity participation. From a relational empowerment perspective, digital communication is associated with the construction of multidimensional social support structures, including bonding social capital that offers psychological security, bridging social capital that provides diverse social cues, and maintained social capital that reactivates pre-existing relational ties. However, it must be acknowledged that, although the mediating role of social capital reached statistical significance, the indirect effects were modest in magnitude across all three dimensions of social capital, with each estimate below 0.07. This suggests that social capital should be interpreted as a modest but meaningful set of mediators linking digital communication to offline participation, rather than as a dominant explanatory mechanism. Taken together, these forms of social capital may correspond to older adults’ sense of mastery over their multiplex social relationships and may be accompanied by increased confidence to act (104). Concurrently, they enable older adults to experience social connectedness and reciprocal commitments in digital environments (105), thereby strengthening trust in their local communities and encouraging more active involvement in offline public life. Moreover, among the three forms of social capital, bridging social capital showed the strongest association with older adults’ offline activity participation (*β* = 0.265). In the context of digital aging, this finding lent empirical support to Granovetter’s argument concerning the strength of weak ties (106). Whereas strong ties primarily provide emotional comfort and stable support, weak ties cultivated through digital media are more likely to function as bridges, enabling older adults to transcend the homophilous boundaries of their existing networks and forge broader, cross-cutting connections. These weak ties broaden and accelerate information flow within networks, bringing non-redundant information about community activities and opportunities for participation directly to older adults, which correlates with expanded access to wider social spaces (107, 108).

It should be noted that, as explicitly explained to participants during the study briefing, the construct of digital media in this study extended well beyond WeChat, despite its prominence among Chinese older adults (20). Rather, it referred to a broader media ecology encompassing social media, online communities, service-oriented platforms, wearable devices, and related digital forms. Correspondingly, digital communication was defined as a broad range of interactive practices enacted through these media, ranging from one-to-one messaging to asynchronous forms of platform-based social interaction. Consequently, the associative pathways identified in this study reflected the generalized functional mechanisms of digital communication rather than platform-specific effects tied to any single application. Building on these findings, we recommend that older adults actively leverage digital media as a multifaceted tool for relational empowerment. Engaging in diverse digital practices may facilitate the accumulation of social capital, particularly by fostering bridging ties grounded in shared interests and local community bonds (109). At the same time, community organizations should proactively utilize digital channels, such as online groups, to cultivate digital public spaces that expand affordances for social connection. By ensuring the continuous delivery of appealing cultural and recreational information, such initiatives may lower barriers to cross-cutting interaction and support the gradual building of social capital. These efforts are especially important for older adults whose social circles have contracted following retirement.

### 5.3 Poor get richer: the moderating effect of social self-efficacy

The findings indicated that social self-efficacy negatively moderated the associations between digital communication and the three forms of social capital, thereby addressing the research question of this study. In contrast to prior evidence based primarily on youth and migrant samples (110–112), this analysis, situated within the context of digital aging, provided support for the social compensation effect of digital media and underscored the potential of digital technologies to alleviate deficits in individuals’ social resources and to narrow intergroup inequalities in social connectedness (113, 114). This study indicates that digital communication enables older adults who are prone to social anxiety in face-to-face interactions to reduce the psychological burdens associated with synchronous offline encounters. By offering an alternative pathway that allows them to compensate for limitations in offline social functioning and more effectively mobilize social resources, digital communication enables these older adults to achieve greater marginal gains in social capital accumulation (115, 116). By contrast, older adults with higher levels of social self-efficacy typically possess more advanced social skills and are adept at securing social support and resources through interpersonal interactions (117, 118). For this group, the ceiling effect constrains the incremental benefits of digital communication. This finding indicates that in advancing age-friendly digital initiatives, technology designers and community practitioners should move beyond the focus on device access and basic operational training. Instead, greater emphasis should be placed on enhancing older adults’ sense of digital competence, shifting the focus of intervention from the question of “whether they can operate the technology” to “whether they are able to engage in meaningful digitally mediated interactions.” Attention should be directed toward socially withdrawn older adults. Inclusive technology design can be leveraged to help these older adults overcome the barriers posed by psychological resistance and limited digital skills. Such efforts may represent an important pathway for enabling this group to break out of social isolation and move toward active aging.

### 5.4 Limitations and future research

It is necessary to acknowledge several limitations of this study. First, the cross-sectional design limited our ability to draw robust causal inferences about the relationships among the study variables. It is also possible that older adults who were already more socially active or more engaged in offline activities were more likely to use digital communication tools. Thus, the direction of the association cannot be firmly established, and reciprocal dynamics may also exist. Future work should adopt longitudinal or experimental designs to more rigorously establish temporal sequencing and clarify the direction of causality. Second, with respect to sampling and recruitment, this study primarily relied on in-person surveys conducted in public settings, which might have overrepresented older adults who were mobile and socially active. In addition, individuals aged 85 and above were excluded, and the sample was drawn primarily from urban areas. These features may limit the generalizability of the findings. In particular, the extent to which these findings apply to older adults with greater physical and psychological vulnerability and a higher risk of social isolation remains to be examined. Future research should adopt more inclusive recruitment strategies, such as combining household-based surveys with telephone interviews, to strengthen the validity and improve sample representativeness. Third, to reduce respondent burden and enhance comprehension, we adopted a careful item-reduction strategy for several measurement scales. Pilot testing suggested that this approach helped reduce inattentiveness associated with overly long questionnaires. Prior research also indicates that, in specific research contexts, abbreviated measures can still demonstrate acceptable construct validity (119, 120). Nevertheless, future studies should consider using more comprehensive instruments for key constructs and corroborating measurement evidence across multiple data sources, thereby better preserving construct richness and further strengthening measurement reliability. Fourth, as this study relied on self-reported survey data, the findings may have been subject to social desirability bias, with participants potentially presenting overly favorable accounts of their experiences. Future research could complement survey data with in-depth interviews or diary methods to generate thick descriptions of older adults’ digital lives. Finally, the present findings should be interpreted within a specific sociocultural context. Compared with cultural settings that emphasize individual autonomy, the relationally oriented context in which many urban older adults in China are embedded may render interpersonal interdependence and reciprocal ties more salient (121). In this sense, the mediating role of social capital observed in the present study may, at least in part, reflect the influence of this context. However, whether this mediating mechanism operates similarly in other cultural settings remains to be examined in future research.

## 6 Conclusion

Drawing on a digital empowerment perspective, this study found that older adults’ digital communication use was positively associated with their offline activity participation. This association was mediated by bonding, bridging, and maintained social capital, thereby providing empirical support for the network enhancement effect of digital media among older adults. Notably, social self-efficacy functioned as a negative moderator. Digital communication contributed to greater marginal gains to social capital accumulation among older adults with lower social self-efficacy, corroborating the social compensation hypothesis in the context of digital media. Based on these findings, the study recommends that age-friendly digital initiatives prioritize enhancing older adults’ perceived digital benefits by building mechanisms that link online connectedness to offline participation. Specifically, these initiatives should focus on older adults with lower self-efficacy and support them through more inclusive interface and interaction design, together with sustained social support. Such efforts may be associated with a more equitable distribution of digital dividends and with higher-quality social integration in later life.

## Data Availability

The raw data supporting the conclusions of this article will be made available by the authors, without undue reservation.
